# The Yin and Yang actions of North American ginseng root in modulating the immune function of macrophages

**DOI:** 10.1186/1749-8546-6-21

**Published:** 2011-05-27

**Authors:** Chike Godwin Azike, Paul Abrahams Charpentier, Jirui Hou, Hua Pei, Edmund Man King Lui

**Affiliations:** 1 Ontario Ginseng Innovation and Research Consortium, the University of Western Ontario, London, Ontario, N6A 5C1, Canada; 2 Department of Physiology & Pharmacology, Schulich School of Medicine & Dentistry, the University of Western Ontario, London, Ontario, N6A 5C1, Canada; 3 Department of Chemical and Biochemical Engineering, Faculty of Engineering, the University of Western Ontario, London, Ontario, N6A 5C1, Canada

## Abstract

**Background:**

Immuno-modulatory effects of ginseng, including both immuno-stimulatory and immuno-suppressive effects, have been widely reported. This study aims to determine whether the paradoxical immuno-modulatory effect is related to unique phytochemical profiles of different North American (NA) ginseng, namely aqueous (AQ) and alcoholic (ALC) extracts.

**Methods:**

AQ and ALC extracts were prepared and their immuno-bioactivity were studied *in vitro *in murine macrophages (Raw 264.7) through measuring the direct stimulatory production of pro-inflammatory mediator and cytokines as well as the suppression of lipopolysaccharide (LPS)-stimulatory response by the two extracts. Gel permeation chromatography was used to fractionate and isolate phytochemicals for characterization of ginseng extracts.

**Results:**

AQ extract up-regulated the production of nitric oxide (NO), tumour necrosis factor-α (TNF-α) and interleukin-6 (IL-6) while ALC extract did not. ALC extract but not AQ extract suppressed LPS-induced macrophage NO and TNF-α production. These immuno-stimulatory and suppressive effects were exhibited at similar extract concentrations. Moreover, the macrophage-stimulating activity of the AQ extract was inhibited in the presence of ALC extract. Fractionation of AQ extract revealed the presence of two major peaks at 230 nm with average molecular weights of 73,000 and 37,000 Da. The first fraction had similar elution volume as the crude polysaccharide (PS) fraction isolated from the AQ extract, and it was the only bioactive species. Parallel fractionation study of ALC extract yielded similar elution profiles; however, both sub-fractions were devoid of PS. Fraction I of the ALC extract suppressed LPS-induced NO production dose-dependently.

**Conclusion:**

ALC extract of NA ginseng, which was devoid of PS, was immuno-inhibitory whereas the AQ extract, which contained PS, was immuno-stimulatory. These extract-related anti-inflammatory and pro-inflammatory effects may be considered as the Yin and Yang actions of ginseng.

## Background

Ginseng is a perennial herb of the Araliaceae family. Asian ginseng (*Panax ginseng *C.A. Meyer, *Renshen*) and NA ginseng (*Panax quinquefolius *L., *Xiyangshen*) are the most commonly used ginseng species. While Asian ginseng has been used for thousands of years as tonic to improve overall health, restore the body to balance, help the body to heal itself and reduce stress [1], the medicinal use of NA ginseng traces back about 400 years ago. Canada is currently the largest producer of NA ginseng [1-3]. Recognized by the Canadian regulatory agency as a natural health product for use as an adaptogen (biological response modifier) [4], NA ginseng is a multi-action herb with a wide range of pharmacological effects on the central nervous system, cardiovascular system and endocrine secretion, reproductive and immune function [5].

Ginseng has influences on both the innate and adaptive immunity. Macrophage-mediated innate immunity is the first line of defence against microbial pathogens and influences the subsequent adaptive immune response. Macrophages kill pathogens and cancer cells directly *via *phagocytosis and indirectly *via *the production of various pro-inflammatory mediators (*e.g. NO*) and cytokines (*e.g. *TNF-α) [6]. However, over production of pro-inflammatory mediators [7] may result in inflammatory diseases and/or tissue injury which are then managed by immune-suppressive agents. Modulation of macrophage function, *e.g. *up-regulation of inflammatory mediator production *in vitro *or suppression of its stimulation by LPS has been used as an experimental tool to evaluate immuno-stimulatory and anti-inflammatory potency of herbal products respectively [8].

Ginseng contains bioactive compounds such as ginsenosides, which are steroidal saponins containing different sugar moieties and possessing different lipid-solubility [5] and polysaccharides (PS) consisting of complex chain of monosaccharides rich in L-arabinose, D-galactose, L-rhamnose, D-galacturonic acid, D-glucuronic acid and D-galactosyl residue [9,10]. Choice of solvents influences the bioactive components in the extracts. This factor is often overlooked by many investigators who focus mainly on biological activities.

Inconsistent immuno-modulatory effects of ginseng have been reported, including both immuno-stimulatory and immuno-suppressive effects [11-22]. The basis for the apparent paradoxical immuno-modulatory effects is unclear but may be attributed to different experimental conditions, *e.g. *choice of extraction solvents.

The objectives of this study are (1) to characterize the apparent paradoxical effects of ginseng by examining the immuno-modulatory effect of AQ and ALC extracts prepared from 4-year-old Ontario grown NA ginseng roots in RAW 264.7 murine macrophage cell line and (2) to explore the characteristics of the immuno-modulatory bioactive substances of ginseng.

## Methods

### Ginseng and its extracts

Four-year-old NA ginseng roots collected in 2007 from five different farms in Ontario, Canada were provided by the Ontario Ginseng Growers Association. Ginseng extracts from each farm were prepared individually and combined to produce composite extracts which were used for phytochemical and pharmacological studies.

### Materials

RAW 264.7 (ATCC TIB 67) murine macrophage cell lines were provided by Dr Jeff Dixon (Department of Physiology and Pharmacology, University of Western Ontario, Canada). Sephadex G75 was purchased from GE healthcare bio-sciences AB (Sweden). Cell culture medium and reagents were purchased from Gibco laboratories (USA). BD OptEIA ELISA kits tumour necrosis factor-α and interleukin-6 (BD Biosciences, USA). LPS from *Escherichia coli *and Griess reagent were purchased from Sigma-Aldrich (USA).

### Preparation of the AQ, ALC and crude PS ginseng extracts

Dried ginseng root samples were shipped to Naturex (USA) for extraction. Samples were ground between ¼ and ½ inch and used to produce the AQ or ALC extract. Briefly, 4 kg ground ginseng roots were soaked three times during five hours in 16 L of water or ethanol/water (75/25, v/v) solution at 40°C. After extraction, the solution was filtered at room temperature. The excess solvent was then removed by a rotary evaporator under vacuum at 45°C. The three pools were combined and concentrated again until the total solids on a dry basis were around 60%. These concentrates were lyophilized with a freeze dryer (Labconco, USA) at -50°C under reduced pressure to produce AQ or ALC ginseng extract in powder form. Yield of the powder extracts from the concentrates was about 66%. The yields of the final extract (mean ± standard deviation of % extractive) from the initial ground root were 41.74 ± 4.92 and 35.30 ± 5.01 for the AQ and ALC extracts respectively.

A solution of AQ extract in distilled water (10 g/10 mL) was prepared, and the crude PS was precipitated by the addition of four volumes of 95% ethanol. The PS fraction was collected by centrifugation at 350 × *g *(Beckman Model TJ-6, USA) for 10 minutes and lyophilized to produce the crude PS extract.

### Chromatography of ginseng extracts

#### High performance liquid chromatography (HPLC) analysis for ginsenoside determination

HPLC analysis on the composition of ginsenosides in AQ and ALC extracts (100 mg/ml methanol) was performed with a Waters 1525 HPLC System with a binary pump and UV detector. A reversed-phase Inspire C18 column (100 mm × 4.6 mm, i.d. 5 μm) purchased from Dikma Technologies (USA) was used for all chromatographic separations. Gradient elution consisted of [A] water and [B] acetonitrile at a flow of 1.3 mL/min as follows: 0 min, 80-20%; 0-60 min, 58-42%; 60-70 min, 10-90%; 70-80 min, 80-20%. Absorbance of the eluates was monitored at 203 nm.

#### Sephadex G-75 chromatography

Five hundred milligrams (500 mg) of AQ or ALC ginseng extract was dissolved in 5 mL distilled water and then fractionated by loading to a calibrated Sephadex G-75 column (47 × 2.5 cm) equilibrated and eluted with distilled water mobile phase at 4°C with a flow rate of 1 mL/min. Absorbance of the eluates was monitored at 230 nm. Fractions (5 mL) were collected and four major fractions (I-IV) were collected and lyophilized to produce four sub-fractions (I-IV) for the study of bioactivity distribution.

#### Size exclusion chromatography for PS analysis

Size exclusion chromatography of AQ, ALC and PS ginseng extract was carried out at 40°C with an AquaGel PAA-200 Series column (8 × 300 mm, PolyAnalytik, USA) connected to a Viscotek (Varian Instruments, USA) gel permeation chromatography system with Omnisec software (version 4.5, Viscotek, USA) for data acquisition. Solutions of AQ, ALC and PS extract (5 mg/mL) were filtered with 0.2 μm nylon filter and used for analysis. Each sample (100 μl) was injected and eluted with 0.05 M sodium nitrate (NaNO_3_) mobile phase at a flow rate of 1 mL/min and monitored using a multiple detectors system for light scattering, refractive index and viscosity. Pullulan polysaccharide reference standard was analyzed as a positive control.

#### Cell culture

Mouse macrophage cell line RAW 264.7 was cultured in Dulbeccos Modified Eagle's Medium supplemented with 10% Fetal Bovine Serum, 25 mM HEPES, 2 mM Glutamine, 100 IU/ml penicillin and 100 μg/ml streptomycin. Cells were seeded in 96-well tissue culture plates at a density of 1.5 × 10^5 ^cells per well and maintained at 37°C in a humidified incubator with 5% CO_2 _and weekly passage and used for experiments at 60-80% confluency.

### Cell treatment

#### Immuno-stimulatory effect

Experiment to evaluate dose-related stimulation of inflammatory mediators profile *in vitro *was carried out by treating and incubating macrophages (1.5 × 10^5 ^cells/well) with 0, 20, 50 and 200 μg/ml of ginseng extracts or 1 μg/mL of LPS (positive control) for 24 hours. The end-points were the 24 hours-production of NO, TNF-α and IL-6 inflammatory mediators.

#### Immuno-suppression of LPS-induced effect

To examine the direct inhibitory effect of ginseng extracts on LPS-stimulated immune function, we pre-treated the macrophages with 0, 10, 50, 100 or 200 μg/ml of ginseng extracts two hours prior to the addition of 1 μg/mL of LPS. The 24-hour cytokine production induced by LPS was determined by measuring NO, TNF-α and IL-6 levels in the culture medium.

#### Suppression of AQ extract-induced macrophage NO stimulation by ALC extract

Production of NO by 1.5 × 10^5 ^macrophages/well in a 96 well-plate induced by 0, 50 and 200 μg/ml of AQ ginseng extract was determined 24 hours after the presence and absence of 200 μg/ml ALC ginseng extract.

#### Quantification of NO, TNF-α and IL-6

TNF-α and IL-6 concentrations in supernatants from cultured cells were analyzed with ELISA. Samples were evaluated with mouse cytokine-specific BD OptEIA ELISA kits (BD Biosciences, USA) according to the manufacturer's protocol. NO production was analyzed as accumulation of nitrite in the culture medium. Nitrite in culture supernatants was determined with Griess reagent (Sigma-Aldrich, USA). Briefly, 50 μL of culture supernatant from each sample were transferred to wells of a 96-well U-bottom microtiter plate, 50 μL Griess reagent (containing 0.5% sulfanilic acid, 0,002% N-1-naphtyl-ethylenediamine dihydrochloride and 14% glacial acetic acid) was then added. The absorbance at 550 nm wavelength was measured using Multiskan Spectrum microplate reader (Thermo Fisher Scientific, Finland) with SkanIt software (version 2.4.2, Thermo Fisher Scientific, Finland). Sample nitrite concentrations were estimated from a sodium nitrite standard calibration curve.

### Statistical analysis

Each cell culture experiment was performed at least three separate times. All statistical analyses were performed with GraphPad prism 4.0a Software (GraphPad Software Inc., USA). Data were presented as the mean ± standard deviation (SD) of triplicates from three independent experiments. Data sets with multiple comparisons were evaluated by one-way analysis of variance (ANOVA) with Dunnett's *post-hoc *test. *P *< 0.001 was considered to be statistically significant.

## Results

### Phytochemical characteristics of the AQ and ALC ginseng extracts

HPLC analysis of the AQ and ALC ginseng extracts showed significant differences in the total ginsenoside (Rb_1_, Re, Rc, Rd, Rg_1 _and Rb_2_) content and profiles. ALC extract contained over twice as much amount of total ginsenosides as the AQ extract, namely 28.25% *vs*. 13.87% dry weight of extract. Rb_1 _and Re were the two most predominant ginsenosides in both extracts but the Rb_1_/Re ratio was higher in the ALC extract, namely 1.8 *vs*. 1.1. No detectable levels of Rh_1 _were measured.

### Immuno-stimulatory effect of the AQ and ALC ginseng extracts in macrophages in vitro

**Figure 1:**
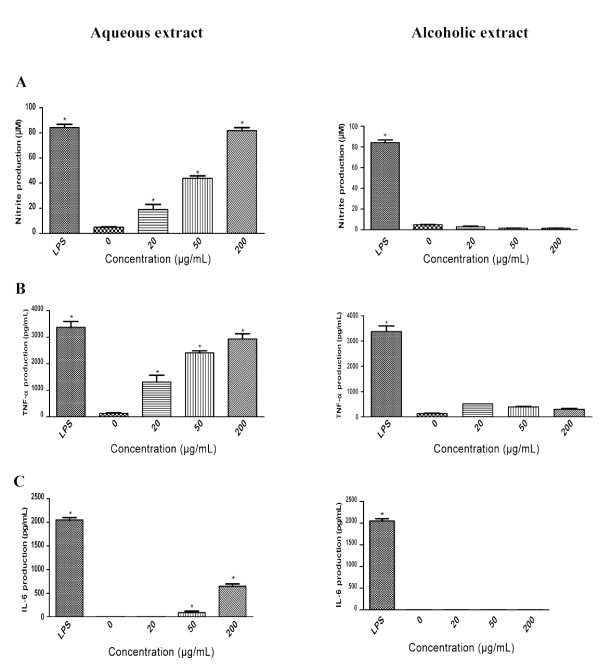
**Immuno-stimulatory effects of the AQ and ALC ginseng extracts on 24 hours macrophage production of (A) NO, (B) TNF-α and (C) IL-6**. Murine macrophages (RAW 264.7 cells) were treated with or without AQ and ALC ginseng extracts (20, 50, 200 μg/ml), LPS (1 μg/ml) for 24 hours and the culture supernatants were analysed for NO and TNF-α/IL-6 by Griess reaction assay and ELISA respectively. Three independent experiments were performed and the data were shown as mean ± SD. Datasets were evaluated by ANOVA. * Values *P *< 0.001 compared to the untreated (vehicle) control were statistically significant.

Evaluation of the immuno-stimulatory effect of the ginseng extracts on RAW 264.7 murine macrophages revealed that exposure to 20-200 μg/mL of AQ extract significantly up-regulated macrophage production of NO, TNF-α and IL-6 compared to untreated control in a concentration-dependent manner (Figure 1). The responses to 200 μg/mL of AQ extract in NO and TNF-α production were similar to the maximum stimulatory response induced by 1 μg/mL of LPS. Moreover, the magnitude of maximum stimulatory response pertaining to NO and TNF-α (as a % of the positive control) was much greater that of IL-6. By contrast, the ALC extract had no apparent immuno-stimulatory effect (Figure 1).

### Effect of the AQ and ALC ginseng extracts on LPS-stimulated production of NO and TNF-α in macrophages in vitro

**Figure 2:**
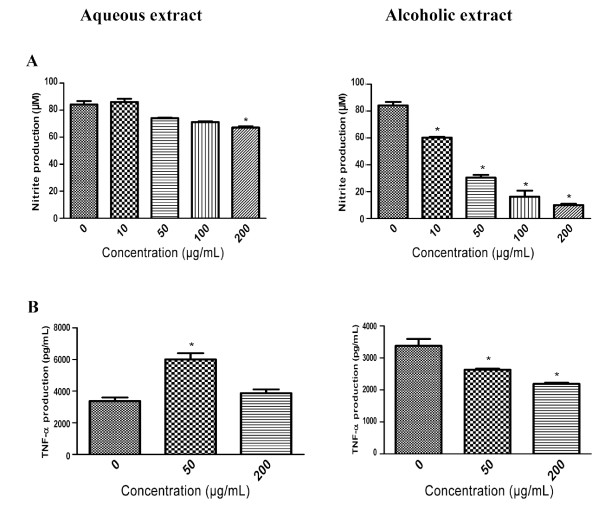
**Effect of the AQ and ALC ginseng extracts on LPS-stimulated 24 hours macrophage production of (A) NO and (B) TNF-α**. Murine macrophages (RAW 264.7 cells) were pre-treated with without the AQ and ALC ginseng extracts (50, 200 μg/ml), for two hours after which LPS 1 μg/ml was added; and 24 hours later the NO and TNF-α contents of the culture supernatants were determined by Griess reaction assay and ELISA, respectively. Three independent experiments were performed and the data were shown as mean ± SD. Datasets were evaluated by ANOVA. * Values *P *< 0.001 compared to the LPS positive control were statistically significant.

Figure 2 showed the influence of ginseng extract treatment on LPS-stimulated NO and TNF-α production in macrophages. LPS stimulated 24-hour production of NO markedly, which was significantly suppressed in the presence of 20-200 μg/ml of the ALC extract in a dose-dependent manner (Figure 2). This inhibitory effect appeared to be extract-specific as the AQ extract was marginally effective and only at high concentrations (Figure 2). Figure 2 also showed that the influence of ginseng was cytokine-specific, *i.e. *the magnitude of inhibition by ALC extract was much smaller with respect to TNF-α production. Moreover, the AQ extract had either no inhibitory effect at high concentration or additive effect at low concentration.

### Suppression of the AQ ginseng extract-induced immuno-stimulation by the ALC ginseng extract

**Figure 3:**
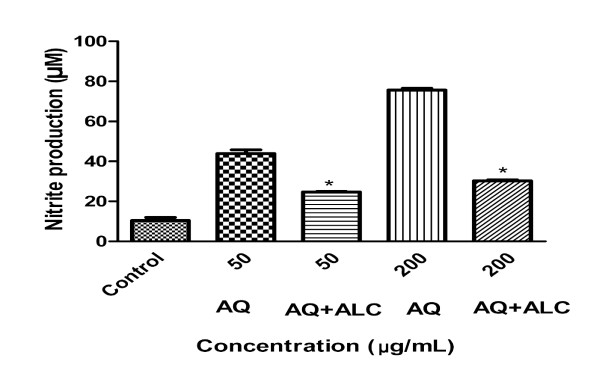
**ALC ginseng extract suppressed up-regulation of macrophage NO production by the AQ ginseng extract**. Murine macrophages (RAW 264.7 cells) were pre-treated with or without the 200 μg/ml ALC ginseng extracts (50, 200 μg/ml), for two hours after which the AQ ginseng extract (50, 200 μg/ml) was added, and the NO contents of the culture supernatants were determined by Griess reaction assay 24 hours later. Three independent experiments were performed and the data were shown as mean ± SD. Datasets were evaluated by ANOVA. * Values *P *< 0.001 were statistically significant.

To further study the apparent extract-specific paradoxical immuno-modulatory effects of ginseng, we carried out an experiment to determine whether the immuno-stimulation induced by the AQ extract could be suppressed by concurrent treatment with the ALC extract. The dose-related up-regulation of NO production in macrophages by the AQ extract was reduced by 50-65% with exposure to equivalent concentrations of the ALC extract (Figure 3).

### Immuno-stimulatory and immuno-suppressive components of the AQ and ALC ginseng extracts

**Figure 4:**
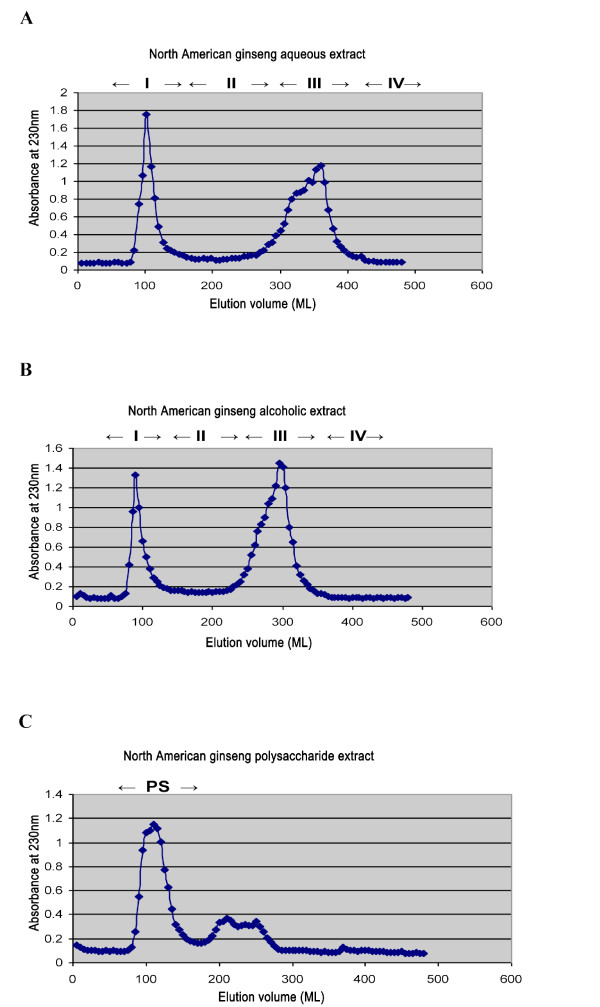
**Sephadex G-75 (47 × 2.5 cm) chromatographic fractionation of the (A) AQ, (B) ALC and (C) PS extracts of ginseng**. Column was loaded with 500 mg of extract, and then eluted with distilled water at flow rate of 1 mL/min. The y-axis is the absorbance at 230 nm while the x-axis represents the elution volume (mL).

To further study the apparent extract-specific paradoxical immuno-modulatory effects of ginseng, we examined the extract-specific bioactive compounds that mediated these effects. Gel filtration of the AQ extract on a Sephadex G-75 column resulted in the appearance of two major peaks (Fractions I and III) based on the absorbance at 230 nm (Figure 4A). The estimated average molecular weights of Fractions I and III were about 73,000 and 37,000 Da respectively; and their yield accounted for 28% and 40% by dry weight of the AQ extract respectively. Since PS of ginseng possesses an immuno-stimulatory effect [9,10,13,23], the crude PS fraction was isolated from the AQ extract by alcohol (40%) precipitation (with a yield of 10% by weight) and was subjected to similar chromatographic procedure for comparison. As shown in Figures 4A and 4C, the major PS peak had a similar elution volume as Fraction I of the AQ extract.

**Figure 5:**
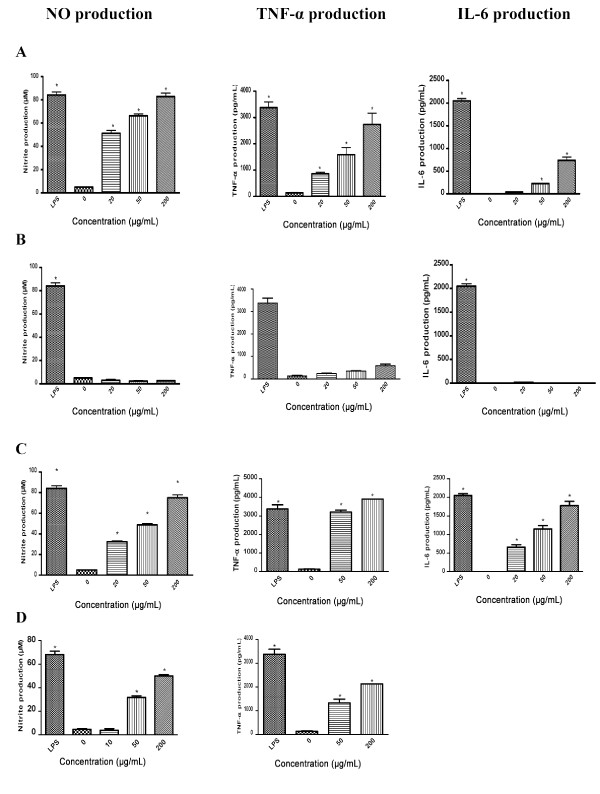
**Immuno-stimulatory effect of Fraction I and III of the AQ, PS extracts of ginseng and Cold-Fx**. Murine macrophages (RAW 264.7 cells) were treated with Fraction I and III of AQ ginseng extract, PS extract of ginseng and Cold-Fx. (0, 20, 50, 200 μg/ml), LPS (1 μg/ml) for 24 hours and the NO, TNF-α and IL-6 contents of the culture supernatants were determined. Three independent experiments were performed and the data were shown as mean ± SD. Datasets were evaluated by ANOVA. *Values *P *< 0.001 compared to the untreated (vehicle) control were statistically significant.

Figure 5 showed the data concerning the stimulation of cytokine production in macrophages by these fractions. Stimulatory activity of Cold-Fx, a commercial natural health product with well established immuno-stimulatory activity [9,10], was included as a reference. The immuno-stimulatory activity with respect to NO and TNF-α production was associated only with macromolecules of Fraction I but not Fraction III and the potency of the former was similar to the PS extract and was better than Cold-Fx. Fraction I was less active than the PS extract in terms of IL-6 production. Since PS and Fraction I corresponded to 10% and 28% of the AQ extract by weight, it appeared that these isolated chemical constituents could only account for part of the observed immuno-stimulatory activities of the AQ extract on the basis of the difference in their immuno-stimulatory potency.

**Figure 6:**
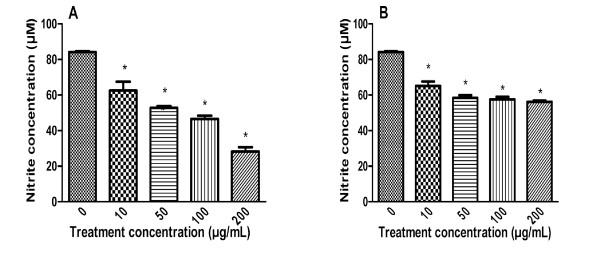
**Effect of Fractions I and III of the ALC extract on LPS-stimulated 24 hours macrophage production of NO**. Murine macrophages (RAW 264.7 cells) were pre-treated with or without the AQ and ALC extracts (10, 50, 100, 200 μg/ml) for two hours after which LPS (1 μg/ml) was added, and the NO content of the culture supernatants were determined by Griess reaction assay 24 hours later. Three independent experiments were performed and the data were shown as mean ± SD. Datasets were evaluated by ANOVA. * Values *P *< 0.001 compared to the LPS positive control were statistically significant.

Fractions I (10%) and III (64%) obtained from Sephadex chromatographic profile of the ALC extract contained no immuno-stimulatory activity (data not shown). Fraction I was not affected by treatment with 40% ethanol (data not shown). This observation was consistent with the lack of PS in the ALC extract. Figure 6 indicated that Fraction I of ALC extract was particularly more active than Fraction III: causing significant and dose-dependent reduction in 24-hour NO production by macrophages induced by 1 μg/mL LPS. Moreover, treatment with 200 μg/ml of Rb_1 _and Rg_1 _did not have significant effects on LPS-induced 24-hour NO production in macrophages (data not shown).

**Figure 7:**
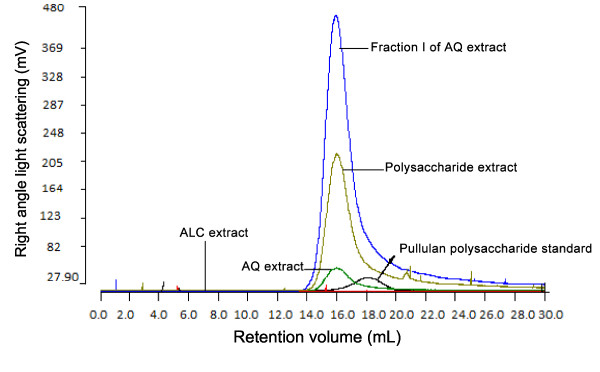
**Identification of polysaccharides in Fraction I of the AQ extract, PS extract, AQ extract and ALC extract of ginseng by size exclusion chromatography**. 100 μL of 5 mg/mL sample or 2.4 mg/mL standard was injected and eluted with 0.05 M NaNO_3 _mobile phase, this was monitored with right angle light scattering detector at 1 mL/min flow rate. Pullulan polysaccharide was used as a reference standard. The y-axis is the detector response (mV) while the x-axis represents the retention volume (mL). No signal was detected with the ALC extract, suggesting the absence of polysaccharides in this extract.

In view of the similarity in the Sephadex G-75 profile of the AQ and ALC extracts, we used more specific chromatographic technique to differentiate the macromolecular constituents from the two extracts. Light scattering data in Figure 7 showed the presence of PS in Peak I of AQ extract on the basis of its similarity to the polysaccharide reference and the crude PS fraction isolated from ginseng. By contrast, Peak I of the ALC extract contained no detectable PS.

## Discussion

The present study delineated the paradoxical immuno-modulatory effect of ginseng and provided a basis for explaining the apparently contradictory reporting in the literature. The observed extract-specific immuno-stimulatory and immuno-suppressive effects were described independently by a number of investigators who examined the activity of the aqueous [11-15] or alcoholic [16-21] extracts of ginseng. Moreover, there was a pattern of association of immuno-stimulation and immuno-suppressive activities with aqueous [11-15] and alcoholic [16-21] extracts respectively, with the exception of a study showing an aqueous extract to possess immunosuppressive effect [22]. In light of the observed paradoxical effects and similarity in the yield (% extractive of 41.4 and 35.3) and potency of the AQ and ALC extracts (Figure 1 and 2), we consider the extract-specific inhibitory and stimulatory effect on macrophage function reported in the present study as the Yin and Yang actions of ginseng. This concept was considered as an extension of the Yin and Yang actions of ginseng proposed by other investigators on angiogenesis [24] and cancer cell proliferation [25,26].

Findings on the macrophage-stimulating effect of NA ginseng (Figure 1) have provided new information on the immuno-stimulatory property of ginseng in term of its cytokine specificity and dose dependency, which also reflected on the specific pharmacological basis of its biological activity. In a separate study we have also demonstrated that the same AQ extract stimulated inflammatory cytokines (IL-1β, IL-6, TNF-α) as well as IL-10 response by human peripheral blood mononuclear cells (PBMC) [27]. Moreover, the changes reported above were not due to LPS contamination of the extracts as documented by Limulus test and direct LPS assay. The potency of the AQ extract was significant in that its stimulatory activities per unit weight is either similar or better than that of Cold-Fx, a licensed Canadian Natural Health Product enriched in polysaccharides for the management of common cold and upper respiratory infections [9,10].

While ginseng is generally regarded as an immuno-booster or adaptogen [1], a recent study reported that Rb_1 _ginsenoside purified from an alcoholic ginseng extract induced an anti-arthritic effect in an animal model [28]. The present study also showed that the inhibitory effect of the ALC extract could be extended to the stimulation induced by the AQ extract, suggesting that both immuno-stimulatory and immuno-suppressive components were present. Use of certain solvent systems may lead to an inactive extract. Although the magnitude of the inhibition on LPS-induced NO response by the ALC extract was quite significant, the suppressive effect was highly specific to the cytokine involved since the TNF-α response was not affected. Furthermore, this study showed that the ALC extract did not affect inflammatory response to LPS in monocytes and T-cells isolated from human PBMC [27]. Further studies are required to address the target cell and signalling pathways specificity of the ALC extract.

Many medicinal plants possess immuno-stimulatory activity and polysaccharides have been recognized as the primary bioactives [23]. In this study, relative abundance of PS (Fraction I) in the AQ extract (Figure 4) was well correlated with its immune-stimulatory activity (Figure 5). Plant bioactive polysaccharides were reported to have molecular weights ranging from 10,000 to 150,000 Da [9,10]. The estimated molecular weight of the ginseng immuno-stimulatory PS reported in our study was within this range. Figures 1 and 5 indicated that the total macrophage-stimulating activity of the AQ extract was not solely due to Fraction I and/or the PS fraction since the immuno-stimulatory effects of the AQ extract were more potent than those of the PS or Fraction I. It is possible that some of the bioactive material was lost during isolation or fractionation procedures. It has been suggested that ginsenosides may be involved immuno-suppression [16,19,20], which is consistent with the higher total ginsenoside levels with the ALC extract. However, this study showed that ginsenosides Rb_1 _and Rg_1 _were not active and that the inhibitory activity was associated with macromolecular Fraction I (molecular weight of 66,000-82,000 Da). Figure 7 indicated that it was not PS. This finding should provide new directions for researchers exploring anti-inflammatory agents in ginseng.

Findings on the extract-specific immuno-modulatory effect have significant implications in the safety, manufacturing, production, development and regulation of products based on ginseng extracts. It is unknown whether the use of organic solvents or the extraction protocol may influence the potency and characteristics of the extracts of other ginseng species. It is imperative to carry out a systematic analysis of the physiochemical characteristics of various ginseng extracts to determine how these parameters may influence their immuno-modulatory properties. The present study provides a lead for identifying immuno-bioactive constituents of ginseng.

## Conclusion

ALC extract of NA ginseng, which was devoid of PS, was immuno-inhibitory whereas the AQ extract, which contained PS, was immuno-stimulatory. These extract-related anti-inflammatory and pro-inflammatory effects may be considered as the Yin and Yang actions of ginseng.

## Abbreviations

ALC: Alcoholic; AQ: Aqueous; HPLC: High Performance Liquid Chromatography; IL-6: Interleukin-6; LPS: Lipopolysaccahride; NA: North America; NO: Nitric Oxide; PBMC: Peripheral Blood Mononuclear Cells; PS: Polysaccharides; TNF-α: Tumor Necrosis Factor-alpha

## Competing interests

The authors declare that they have no competing interests.

## Authors' contributions

EMKL conceived the study design, interpreted the data and wrote the manuscript. He also acquired research funding and resources. CGA carried out the experiments evaluated the immuno-modulating effects and drafted the manuscript. PAC collaborated with PolyAnalytik London Ontario, Canada to perform the gel permeation chromatography of ginseng extracts. JH performed the HPLC analysis of ginseng extracts. HP lyophilized the ginseng extracts and maintained the murine macrophages (Raw 264.7) culture. All authors read and approved the final version of the manuscript.

## References

[B1] Angelova N Kong HW van der Heijden R Yang SY Choi YH Kim HK Wang M Hankemeier T van der Greef J Xu G Verpoorte R Recent methodology in phytochemical analysis of ginseng Phytochem Anal 2008 19 2 16 10.1002/pca.1049 18058794

[B2] Borchers AT Keen CL Stern JS Gershwin ME Inflammation and native American medicine: the role of botanicals Am J Clin Nutr 2000 72 339 47 10919925 10.1093/ajcn/72.2.339

[B3] Agriculture and Agri-Food Canada, Overview of the Canadian Special Crops Industry - Ginseng http://www.ats-sea.agr.gc.ca/can/4752-eng.htm#j

[B4] Health Canada Drugs and Health Products, Natural Health Product Monograph Panax Ginseng http://www.hc-sc.gc.ca/dhp-mps/alt_formats/pacrb-dgapcr/pdf/prodnatur/applications/licen-prod/monograph/mono_panax_ginseng-eng.pdf

[B5] Attele AS Wu JA Yuan CS Ginseng pharmacology; multiple constituents and multiple actions Biochem Pharmacol 1999 58 1685 1693 10.1016/S0006-2952(99)00212-9 10571242

[B6] Wood PJ The immune system: recognition of infectious agents Anaesth Intensive Care Med 2006 7 179 180 10.1383/anes.2006.7.6.179

[B7] Bondeson J The mechanisms of action of disease-modifying antirheumatic drugs: a review with emphasis on macrophage signal transduction and the induction of proinflammatory cytokines Gen Pharmacol 1997 29 127 150 9251892 10.1016/s0306-3623(96)00419-3

[B8] Lee MY Park BY Kwon OK Yuk JE Oh SR Kim HS Lee HK Ahn KS Anti-inflammatory activity of (-)-aptosimon isolated from Daphne genkwa in RAW264.7 Int Immunopharmacol 2009 9 878 885 10.1016/j.intimp.2009.03.012 19328870

[B9] Biondo PD Goruk S Ruth MR O'Connell E Field CJ Effect of CVT-E002™ (COLD-fX^®^) versus a ginsenoside extract on systemic and gut-associated immune function Int Immunopharmacol 2008 8 1134 1142 10.1016/j.intimp.2008.04.003 18550018

[B10] Wang M Guilbert LJ Li J Wu Y Pang P Basu TK Shan JJ A proprietary extract from North American ginseng (Panax quinquefolium) enhances IL-2 and IFN-gamma productions in murine spleen cells induced by Con-A Int Immunopharmacol 2004 4 311 315 10.1016/j.intimp.2003.12.002 14996422

[B11] Jie YH Cammisuli S Baggiolini M Immunomodulatory effects of Panax Ginseng C.A. Meyer in the mouse Agents Actions 1984 15 3 4 10.1007/BF01972376 6084415

[B12] Friedl R Moeslinger T Kopp B Spieckermann PG Stimulation of nitric oxide synthesis by the aqueous extract of Panax ginseng root in RAW 264.7 cells Br J Pharmacol 2001 134 1663 1670 10.1038/sj.bjp.0704425 11739242 PMC1572905

[B13] Assinewe VA Arnason JT Aubry A Mullin J Lemaire I Extractable polysaccharides of Panax quinquefolius L. (North American ginseng) root stimulate TNF-α production by alveolar macrophages Phytomedicine 2002 9 398 404 10.1078/09447110260571625 12222658

[B14] Lee JW Takano-Ishikawa Y Watanabe J Kobori M Tsushida T Yamaki K Effect of ginsenosides and red ginseng water extract on tumor necrosis factor-α production by rat peritoneal macrophages Food Sci Technol Res 2002 8 300 303 10.3136/fstr.8.300

[B15] Zhou DL Kitts DD Peripheral blood mononuclear cell production of TNF-α in response to North American ginseng stimulation Can J Physiol Pharmacol 2002 80 1030 1033 10.1139/y02-116 12450071

[B16] Lee DC Yang CL Chik SC Li JC Rong JH Chan GC Lau AS Bioactivity-guided identification and cell signalling technology to delineate the immunomodulatory effects of Panax ginseng on human promonocytic U937 cells J Transl Med 2009 34 1 10 10.1186/1479-5876-7-34 PMC2689162 19442267

[B17] Park JS Park EM Kim DH Jung K Jung JS Lee EJ Hyun JW Kang JL Kim HS Anti-inflammatory mechanism of ginseng saponins in activated microglia J Neuroimmunol 2009 209 40 49 10.1016/j.jneuroim.2009.01.020 19232442

[B18] Li J Ichikawa T Nagarkatti P Nagarkatti M Hofseth LJ Windust A Cui T American ginseng preferentially suppresses STAT/iNOS signaling in activated macrophages J Ethnopharmacol 2009 125 145 150 10.1016/j.jep.2009.05.032 19505555 PMC2790430

[B19] Rhule A Rase B Smith JR Shepherd DM Toll-like receptor ligand-induced activation of murine DC2.4 cells is attenuated by Panax notoginseng J Ethnopharmacol 2008 28 179 186 10.1016/j.jep.2007.11.019 PMC2265075 18164154

[B20] Rhule A Navarro S Smith JR Shepherd DM Panax notoginseng attenuates LPS-induced pro-inflammatory mediators in RAW264.7 cells J Ethnopharmacol 2006 106 121 128 10.1016/j.jep.2005.12.012 16427227

[B21] Liou CJ Huang WC Tseng J Long-term oral administration of ginseng extract modulates humoral immune response and spleen cell functions Am J Chin Med 2005 33 651 661 10.1142/S0192415X05003247 16173538

[B22] Jin UH Park SG Suh SJ Kim JK Kim DS Moon SK Lee YC Park WH Kim CH Inhibitory effect of Panax notoginseng on nitric oxide synthase, Cyclo-oxygenase-2 and neutrophil functions Phytother Res 2007 21 142 148 10.1002/ptr.2018 17128437

[B23] Schepetkin IA Quinn MT Botanical polysaccharides: macrophage immunomodulation and therapeutic potential Int Immunopharmacol 2006 6 317 333 10.1016/j.intimp.2005.10.005 16428067

[B24] Yue PY Mak NK Cheng YK Leung KW Ng TB Fan DT Yeung HW Wong RN Pharmacogenomics and the Yin/Yang actions of ginseng: anti-tumor, angiomodulating and steroid-like activities of ginsenosides Chin Med 2007 2 1 21 10.1186/1749-8546-2-1 17502003 PMC1876803

[B25] Iishi H Tatsuta M Baba M Uehara H Nakaizumi A Shinkai K Akedo H Funai H Ishiguro S Kitagawa I Inhibition by ginsenoside Rg3 of bombesin-enhanced peritoneal metastasis of intestinal adenocarcinomas induced by azoxymethane in wistar rats Clin Exp Metastasis 1997 15 603 611 10.1023/A:1018491314066 9344044

[B26] Tatsuka M Maeda M Ota T Anticarcinogenic effect and enhancement of metastatic potential of BALB/c 3T3 cells by ginsenoside Rh (2) Jpn J Cancer Res 2001 92 1184 1189 11714442 10.1111/j.1349-7006.2001.tb02138.x PMC5926664

[B27] Toth JM Hewson GL Frodermann V Chau LA Azike C Lui E Madrenas J Modulatory effects of ginseng extracts on human innate and adaptive immune responses Proceedings of the 10th International Symposium on Ginseng: 13-16 September, 2010; Seoul, Korea

[B28] Kim HA Kim S Chang SH Hwang HJ Choi YN Anti-arthritic effect of ginsenoside Rb_1 _on collagen induced arthritis in mice Int Immunopharmacol 2007 7 1286 1291 10.1016/j.intimp.2007.05.006 17673143

